# Wetting Behaviors of a Nano-Droplet on a Rough Solid Substrate under Perpendicular Electric Field

**DOI:** 10.3390/nano8050340

**Published:** 2018-05-17

**Authors:** Fenhong Song, Long Ma, Jing Fan, Qicheng Chen, Lihui Zhang, Ben Q. Li

**Affiliations:** 1 School of Energy and Power Engineering, Northeast Electric Power University, Jilin 132012, China; fenhongsong@neepu.edu.cn (F.S.); ml158484015@126.com (L.M.); chenqicheng2010@hotmail.com (Q.C.); 2 Key Laboratory of Special Purpose Equipment and Advanced Processing Technology, Ministry of Education, Zhejiang University of Technology, Hangzhou 310014, China; lhzhang@zjut.edu.cn; 3 Department of Mechanical Engineering, University of Michigan, Dearborn, MI 48128, USA

**Keywords:** electro-wetting, contact angle, rough surface, molecular dynamic simulation

## Abstract

Molecular dynamic simulations were adopted to study the wetting properties of nanoscale droplets on rough silicon solid substrate subject to perpendicular electric fields. The effect of roughness factor and electric field strength on the static and dynamic wetting behaviors of a nano-droplet on a solid surface was investigated at the molecular level. Results show that the static contact angle tends to decrease slightly and show small difference with the increase of roughness factor, while it shows an obvious increase for the ramp-shaped surface because the appearing bottom space reduces the wettability of solid surface. Additionally, under the electric field, a nano-droplet was elongated in the field direction and the equilibrium contact angle increases with the increase of electric field strength. The nano-droplet was completely stretched to be column-shaped at a threshold value of the field. Besides, accompanied by the shape variation of water droplets, the molecular dipole orientations of water molecules experience a remarkable change from a random disordered distribution to an ordered profile because of the realignment of water molecules induced by electric fields.

## 1. Introduction

The wetting of a water droplet on a solid surface plays a critical role in describing the characteristics of solid surfaces in a broad range of technological applications. According to their requirements, some refer to rapid wetting [1,2,3], whereas the others require poor wetting [4,5,6]. Wettability of liquid on a solid surface is determined by numerous factors, such as chemical composition, material properties (e.g., surface energy) and roughness or heterogeneities of the solid substrate, impurities in liquid, and applied external forces (e.g., electric field force) [7]. Generally, the real surface is not perfectly smooth; it is rough either obviously or molecularly. The presence of micro-textured topology and molecular roughness of solid surface is one of the important factors that directly affects the interaction between liquid and substrate. Thus, it is essential to comprehensively explore the microscope mechanism of liquid wetting on rough solid substrate. Recently, both engineers and scientists have paid substantial attention to conducted simulation or experimental studies on the wetting phenomena of a liquid on a rough solid surface [8,9,10,11].

On the micro/nanoscale, molecular dynamic simulation has been proved a very useful tool to study the mechanism of wetting at a molecular level [12,13,14,15]. There are numerous simulation reports for wetting properties of a droplet on geometrically patterned substrates or on molecularly rough surfaces. Chen et al. [16] studied the wettability behaviors of a water droplet on organic-polluted surface pillar structures and found a Wenzel-Cassie transition of critical line when pillar HI ratio is around 1.6. Svoboda et al. [17] found that the roughness on a strongly hydrophilic surface either increases or hardly affects the contact angle, depending on the number of molecules stacking into the nano-grooves, while adding roughness to a weakly wettable surface would transform the surface from hydrophilic to hydrophobic because nano-grooves weaken the solid-liquid interactions. Niu et al. [18] investigated the static properties of a water droplet on a solid surface and the results showed that a water droplet on a pillared silicon surface could transform from the Wenzel state to the Cassie state following vibration of the rough surface. Wang et al. [19] found the Wenzel-Cassie transition was affected by the droplet size and hollow size. It occurred easily with a big droplet and a narrow hollow, while it suffered a lesser effect from the pillar height. Broadly, substrate patterned topology could convert the wettability of the surface according to its practical applications.

Applying an electric field is an effective method for manipulating the wettability of a nano-droplet on a solid surface. Some researchers have conducted simulations and experimental studies on the effect of an electric field on the spreading of droplets on solid substrate and found novel micro phenomena, such as contact angle saturation [20], asymmetrical spreading and asymmetry-to-symmetry transition [21,22,23]. Meanwhile, when the roughness and heterogeneities of the solid surface appear, the electro-wetting process induced by external electric field could be more complicated. The droplet on a rough surface might be in a Cassie state under a weaker applied electric field and it undergoes a Cassie-to-Wenzel wetting transition on the micro-textured (pillar-arrayed) surface after the applied electric field exceeds a critical value [24,25]. However, the statics and dynamics of electro-wetting on molecularly rough surfaces are still far from well understood, especially at the nanoscale. Thus, it is necessary to further investigate the electro-wetting phenomenon on a molecularly rough surface to satisfy the requirements of practical applications. In this paper, the molecular dynamic simulation method is carried out to explore the static and dynamic wetting behaviors of a nano-droplet on a rough silicon solid surface subjected to perpendicular electric fields. Six types of shape are constructed to represent the different roughnesses of the solid surface. Contact angles and some interfacial properties are taken to describe the effect of electric field strength and roughness factors on the complicated electro-wetting.

## 2. Molecular Simulation Model

**Figure 1:**
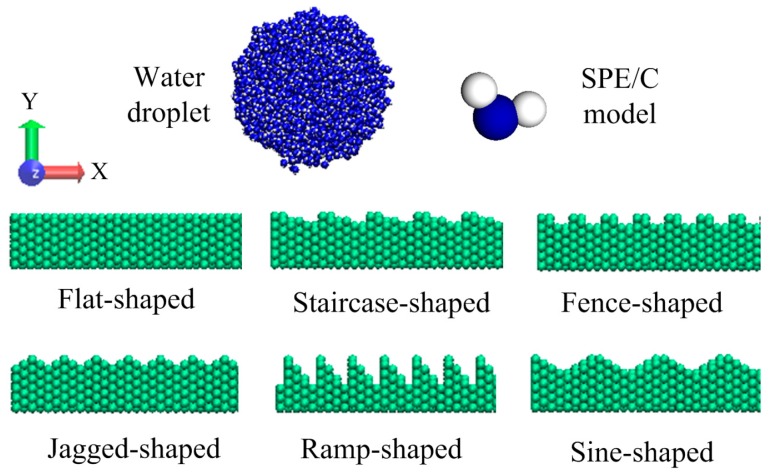
The initial structure of water droplet and substrate with molecularly rough surface.

The initial structure of the nano-droplet and substrate with molecularly rough surface in the simulation system is shown in Figure 1. The Si atoms of rough solid substrate arrange in a diamond cubic lattice, with a lattice constant of 0.543 nm. The size of the substrate is 12 × 1.5 × 12 nm^3^. The atoms of silicon substrate were kept frozen during the molecular dynamic MD simulations to speed up the simulation, as used in the literature [21,23,26]. 2000 water molecules were randomly distributed into a spherical nano-droplet with a radius of 2.5 nm. In fact, droplet size effect on contact angle has been systematically discussed in previous studies [27,28] and we picked this size because it has been found sufficient for studying the wetting phenomenon [21,22,23,29]. The initial distance between the center of the spherical nano-droplet and the substrate surface is 3 nm. A simple point charge/extension (SPC/E) model is chosen for water molecules, which has already been applied successfully to study the wetting phenomena of droplets on solid substrates [21,22,23,26,27,28,29,30]. The intermolecular interactions are calculated as follows. The repulsion and dispersion forces is modeled using the Lennard-Jones (L-J) potential, while the electrostatic interaction using Coulomb’s law,

(1) $$\varphi (r_{ij})=4\varepsilon_{oo}[{\left(\frac{\sigma_{oo}}{r_{oo}}\right)}^{12}-{\left(\frac{\sigma_{oo}}{r_{oo}}\right)}^{6}]+\frac{1}{4\pi \varepsilon_{0}}\sum_{i=1}^{3}\sum_{j=1}^{3}\frac{q_{i}q_{j}}{r_{ij}}$$

where the first L-J potential term is only calculated oxygen-oxygen interactions because the hydrogen atom is too light and the interaction could be omitted. The second term calculates the coulomb force among the charged atoms, where *q_i_* is the charge of atom *i*, *r_ij_* is the distance between atoms *i* and *j*, and *ε*_0_ is the vacuum dielectric constant. Hydrogen bonding in the SPC/E potential is a balance between the L-J oxygen-oxygen forces and the electrostatic hydrogen-oxygen interaction [31].

The intermolecular interactions between water molecules and silicon atoms are calculated by the modified L-J potential function,

(2) $$\varphi_{ls}(r_{ij})=4\varepsilon_{ls}[{\left(\frac{\sigma_{ls}}{r_{ij}}\right)}^{12}-{\left(\frac{\sigma_{ls}}{r_{ij}}\right)}^{6}]$$

where *σ_ls_* and *ε_ls_* can be calculated according to the Lorentz-Berthelot mixing rule [32],

(3) $$\varepsilon_{ls}=\sqrt{\varepsilon_{l}\cdot }\varepsilon_{s},\sigma_{ls}=\left(\sigma_{l}+\sigma_{s}\right)/2.0$$

All the potential parameters and the charges for each type of atoms are given in Table 1.

A smooth cutoff with a cutoff radius of 1.5 nm is used for the short-range L-J pair potential and the Particle-Particle Particle-Mesh (PPPM) method is used to solve the long-range electrostatic interaction. All the MD simulations are carried out using the LAMMPS software package in the NVT ensemble [33]. The initial velocities of atoms were assigned randomly according to the temperature of the simulation system. The Velocity-Verlet algorithm is adopted to solve the Newtonian motion equations for each atom with a time step of 2 fs. The Nosé-Hoover thermostat [34] was applied at each time step to keep the system at a desired temperature 300 K. When an electric field *E* is applied to the system, an additional term representing electric force *f_ie_ = E*·*q_i_* is applied to each atom according to its charge *q_i_*. Firstly, the droplet wetting the silicon solid substrate was equilibrated in the absence of electric field with the simulation runs of 1.2 ns. Then perpendicular electric field *E_y_* with the strength of 0.1, 0.2, 0.5, 0.75, 0.8, 0.9 and 1.0 V/nm is applied to the system to investigate the deformation and wetting behaviors of nanoscale droplet on rough solid surface. After the system reaches a new equilibrium state at 1.2 ns, wetting properties are analyzed to describe the complex wetting behaviors.

## 3. Results and Discussion

### 3.1. Effect of Roughness on Static Contact Angles

Six types of rough surface are constructed to investigate the wetting behaviors of a droplet on a rough solid substrate under an electric field. The front views of roughness are shown in Figure 1 (flat, staircase, fence, jagged, ramp and sine-shaped roughness). In order to describe the degree of roughness, a dimensionless roughness factor (denoted as *r*) is defined as the ratio of the true surface area to the apparent surface area. Obviously, *r* is a number always greater than or equal to 1.0. The exact values of roughness factor and the number of silicon atoms for each type of rough solid substrate are given in Table 2.

**Figure 2:**
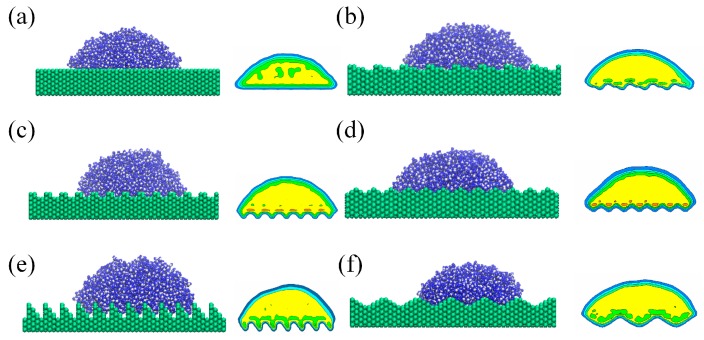
The snapshots in equilibrium state and time-averaged density profiles of nano-droplet on substrate with (**a**) flat-shaped, (**b**) staircase-shaped, (**c**) fence-shaped, (**d**) jagged-shaped, (**e**) ramp-shaped and (**f**) sine-shaped rough surface

**Figure 3:**
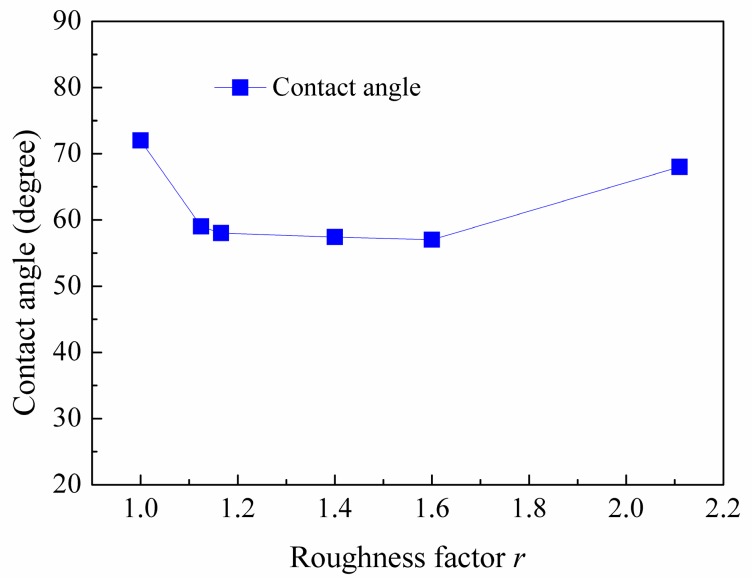
Distribution of the static contact angle of nano-droplet on rough substrate.

Firstly, to check the simulation model, the static contact angle of water nano-droplet on flat silicon surface (*r* = 1.00) in the absence of electric field is calculated. The result shows that it is 72 ± 0.5 degree and consistent with the experimental data from literature [35,36]. The snapshots in equilibrium state and time-averaged density profiles of water nano-droplet on rough solid surface (*E* = 0.0 V/nm) are shown in Figure 2. It can be seen that water molecules accumulate at the depressions and bulges of the rough surface due to the strong attraction from silicon atoms on the rough area and result in the density near the rough area on the surface being slightly higher than that in the inner droplet. Figure 3 depicts the distribution of the contour line of the water droplet on different rough substrates and the corresponding static contact angles with the increase in roughness factor. The static contact angle is obtained by fitting the droplet contour using the elliptical equation. Clearly, the droplet is in the Wenzel state, except for the case of wetting on the ramp-shaped surface. The static contact angle decreases slightly and shows a small difference with the increase in roughness factor due to the different shape of the roughness. When the nano-droplet wets the ramp-shaped surface (*r* = 2.11), the droplet is in the Cassie-Baxter state and the corresponding contact angle tends to increase. The microscopic mechanism of the wetting of the nano-droplet on the rough surface is the molecular interaction between water molecules and silicon atoms. When the roughness factor increases, the real contact area between droplet and solid becomes bigger leading to a stronger attraction from the solid substrate and thus the contact angle tends to decrease. Meanwhile, when the solid surface is constructed in a ramp shape, molecules accumulate on the depression area before overcoming the rough barrier, accompanied by a small interspace appearing at the bottom. So, the real contact area is smaller than the true surface area and then the surface wettability is reduced with a slight increase of contact angle in this case.

### 3.2. Static Wetting under Electric Field

**Figure 4:**
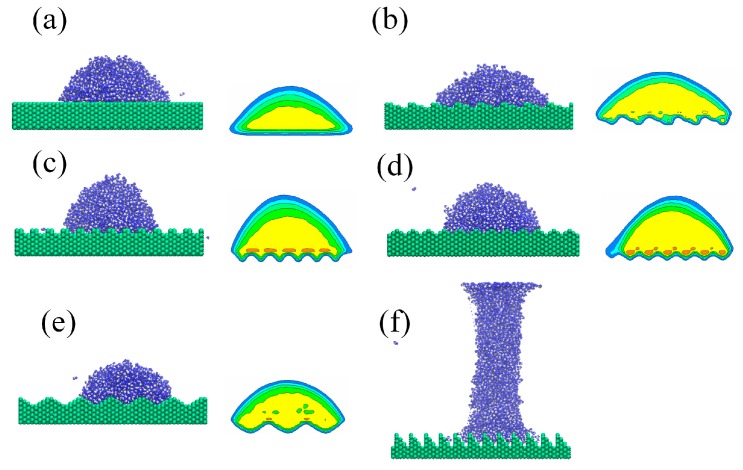
Snapshots and time-averaged density distributions of water nano-droplet in equilibrium state on silicon substrate with (**a**) flat-shaped, (**b**) staircase-shaped, (**c**) fence-shaped, (**d**) jagged-shaped, (**e**) sine-shaped and (**f**) ramp-shaped rough surface (*E_y_* = 0.9 V/nm)

Figure 4 shows the snapshots and time-averaged density distributions of water nano-droplet in the equilibrium state on a rough silicon substrate with different roughnesses subjected to a perpendicular electric field of *E_y_* = 0.9 V/nm. As a classical polar molecule, water dipole rotates under the electric field with the hydrogen atom pointing in the electric field direction and oxygen atom the opposite way. The whole electric field force on the droplet tends to elongate the droplet from both sides along the field direction, while the solid substrate restricts the downward deformation. The greater the surface energy, the stronger the restriction from the surface, and the droplet shows a smaller change induced by electric field. As seen in Figure 4f, the droplet on the ramp-shaped surface is elongated to be a columnar structure because of its small surface energy.

**Figure 5:**
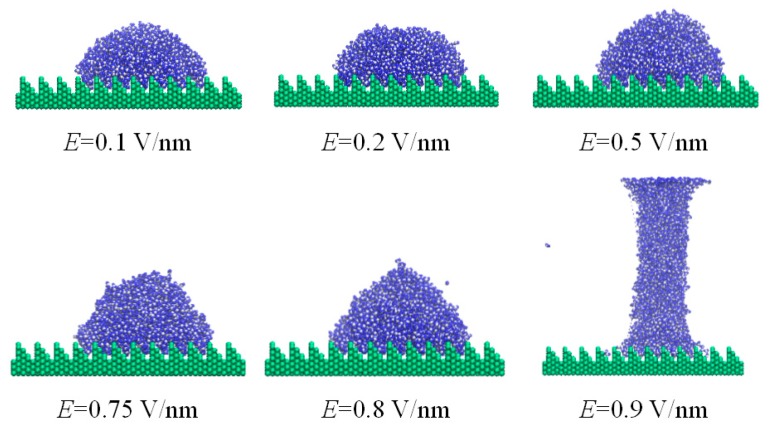
Equilibrium snapshot of water nano-droplet on silicon substrate (ramp-shaped surface) under electric field (*E_y_* = 0.1, 0.2, 0.5, 0.75, 0.8, 0.9 V/nm).

**Figure 6:**
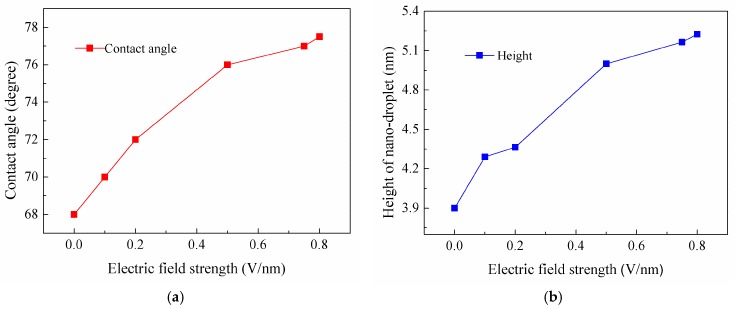
(**a**) Equilibrium contact angle and (**b**) the height of water nano-droplet on silicon substrate (ramp-shaped surface) under different electric fields.

The effect of electric field strength on the static wetting of the nano-droplet on the ramp-shaped surface is also explored. Figure 5 shows the equilibrium snapshots of a water droplet on a ramp-shaped substrate subjected to a perpendicular electric field with strengths of *E_y_* = 0.1, 0.2, 0.5, 0.75, 0.8, 0.9 V/nm. In order to show the wetting behaviors clearly, the corresponding contact angles and the height of droplet in the *y* direction are given in Figure 6. After the electric field is applied to the system, water molecular dipoles rotate to a new equilibrium state with more hydrogen atoms pointing to in the electric field direction. In particular, molecules in the liquid vapor interface of the spherical droplet tend to move upward, and the top liquid-vapor interface of the droplet becomes pointier due to competing effect between surface tension and electric field force. With the increase of electric field strength, the droplet becomes elongated with the height increasing slightly in electric field direction. This indicates that intermolecular forces among water molecules are more dominant than external electric field forces when electric field *E* ≤ 0.8 V/nm. This also could be confirmed in the dipole distribution of water molecules in Section 3.4. Electric field force starts to dominate the movement of polar water molecules with the further increase of electric field and the droplet was elongated to eventually become a column in the case of the ramp shape surface, as shown in Figure 5f. For comparison, more figures for the wetting of nano-droplet on the jagged surface are given in the electronic Supplementary Materials Figure S1.

### 3.3. Dynamic Wetting Process under Electric Field

**Figure 7:**
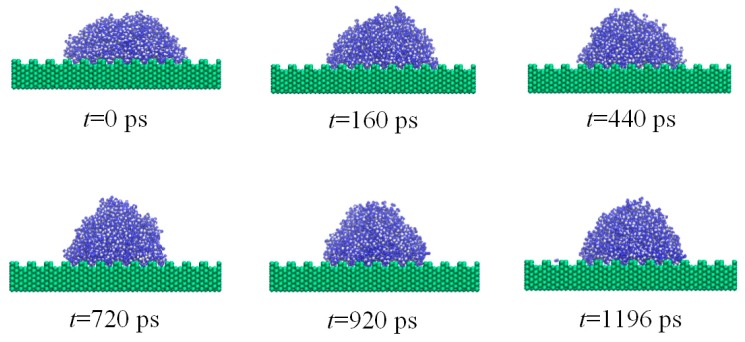
Snapshot of dynamic wetting of the water nano-droplet on silicon substrate (fence-shaped surface) under electric field *E_y_* = 0.9 V/nm.

**Figure 8:**
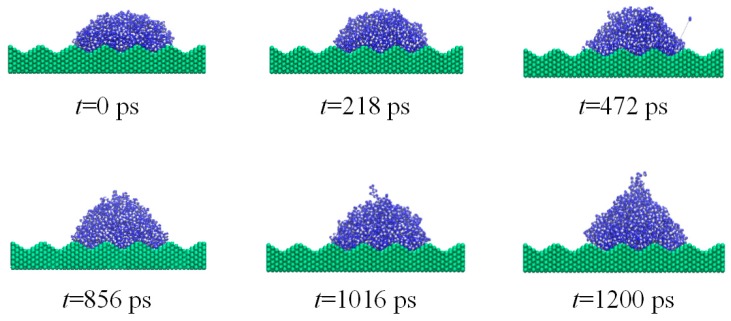
Snapshot of dynamic wetting of the water nano-droplet on silicon substrate (sine-shaped surface) under electric field *E_y_* = 1.0 V/nm.

**Figure 9:**
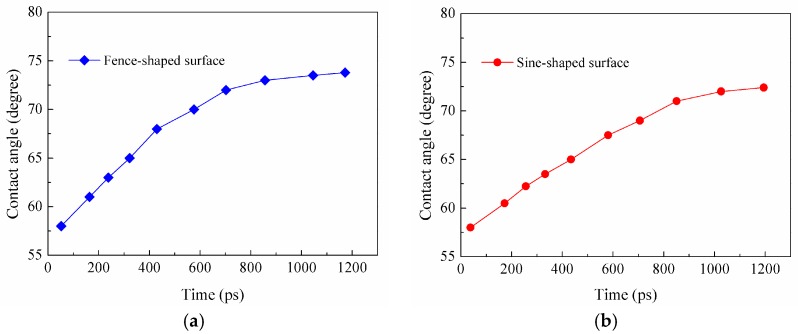
Dynamic contact angles of water nanodroplet on rough silicon substrate under electric field; (**a**) For fence-shaped surface *E_y_* = 0.9 V/nm; (**b**) For sine-shaped surface *E_y_* = 1.0 V/nm.

To further understand the electro-wetting phenomenon of the droplet on the rough solid substrate, the dynamic spreading of a water nano-droplet on a solid substrate with fence-shaped and sine-shaped surfaces are investigated under electric field *E_y_* = 0.9 V/nm and *E_y_* = 1.0 V/nm, respectively. For the fence-shaped substrate, the dynamic wetting, as depicted by several snapshots at different instants of time, is shown in Figure 7 (*E_y_* = 0.9 V/nm). For the sine-shaped substrate, the dynamic wetting of the nano-droplet is given in Figure 8 (*E_y_* = 1.0 V/nm). In addition, the evolution of the dynamic contact angles over time for both rough solid substrates is plotted in Figure 9.

The movements of the water molecule become more complex and turbulent after applying external electric field and are determined by the competition between molecular interaction and electric field force. The electric field tries to rotate water dipoles while molecules around it could restrict them, so molecules on the liquid-vapor interface could move easily because of smaller internal restriction. It could be seen clearly that more water molecules on the top of the water droplet are distributed with hydrogen atoms pointing in the electric field direction and oxygen atoms the opposite way. When the electric field strength is smaller than 0.8 V/nm, the molecule on the solid surface cannot move freely because of the strong attraction from solid substrate. Meanwhile, for the case where *E_y_* > 0.8 V/nm, the movements of water dipoles are dominated by electric field and the water molecules on liquid–vapor interface tend to move upwards. Then, a point is formed on the top of the droplet and the droplet changes to be cone-shaped. As a result, the contact angle decreases. It is apparent in the case of droplet wetting on the sine-shaped surface, as shown in Figure 8f.

In addition, the roughness of the solid substrate, determining the interaction energy between the droplet and the solid surface plays an important role in the dynamic wetting process of the water nano-droplet. As seen in Figure 5f, the droplet on the ramp-shaped surface is completely stretched to become column-shaped under electric field *E*_cr_ = 0.9 V/nm (*E*_cr_ is defined as critical electric field). Meanwhile, for the sine-shaped surface, the droplet is completely stretched under the electric field 1.1 V/nm (as seen in Figure S2). This is all related to the real contact surface area; water molecules contact the sine-shaped surface smoothly and the restriction force to the deformation of the droplet is much larger than others. Therefore, it needs a stronger electric field to be completely stretched.

### 3.4. Distribution of Water Molecular Parameters

#### 3.4.1. Hydrogen Bonds

As discussed above, water molecules realign under the competing force between electric field and intermolecular force. During the rearrangements, hydrogen bonds break up or reform, which could affect the wetting behaviors of a droplet on a rough surface. In MD simulation, there is a criterion of geometrical definition to determine whether a hydrogen bond has formed or not between different water molecules. Firstly, the distance between oxygen atoms is less than 0.35 nm; secondly, the angle between the oxygen-hydrogen bond and oxygen-oxygen direction is less than 30 degrees [37,38]. If these two conditions are satisfied, one hydrogen bond is considered to be formed between them.

**Figure 10:**
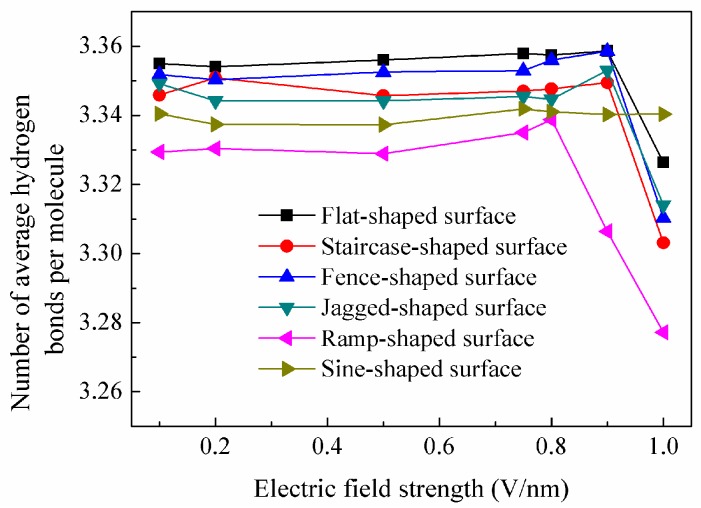
Average number of hydrogen bonds per water molecule under different electric fields.

Theoretically, the maximum number of hydrogen bonds per water molecule is 4.0. At nanoscale, since the specific surface area increases, the number of hydrogen bonds per molecule will decrease and generally is less than 4.0. Figure 10 depicts the average number of hydrogen bonds per water molecule under different electric fields. It could be seen that the average number of hydrogen bonds for the droplet on a specified surface almost stays the same with the increase of the electric field strength before the kick point. This is consistent with the result in the literature [15], in which the number of hydrogen bonds shows a distinct oscillation under different electric fields. As mentioned above, the direction of the dipole tends to follow the direction of the electric field. They are distributed randomly and disorderly when the electric field is smaller than 0.8 V/nm and thus the number of hydrogen bonds is almost unchanged. However, when the electric field exceeds a threshold value, the average number of hydrogen bonds decreases sharply. This is because the electric field strength is strong enough that the disordered dipole distribution becomes ordered and the hydrogen bond nets are destroyed. As a result, the average number of hydrogen bonds decreases rapidly. The average number of hydrogen bonds is largest when a droplet wets a flat silicon surface and smallest on a ramp-shaped surface. This confirms that the shape of the roughness could affect the distribution and arrangement of water molecules and thus has influence on the number of hydrogen bonds per molecule. So, both the electric field and surface roughness have an effect on the distribution of water molecules and the formation of hydrogen bonds.

#### 3.4.2. Water Molecular Dipole Moment

**Figure 11:**
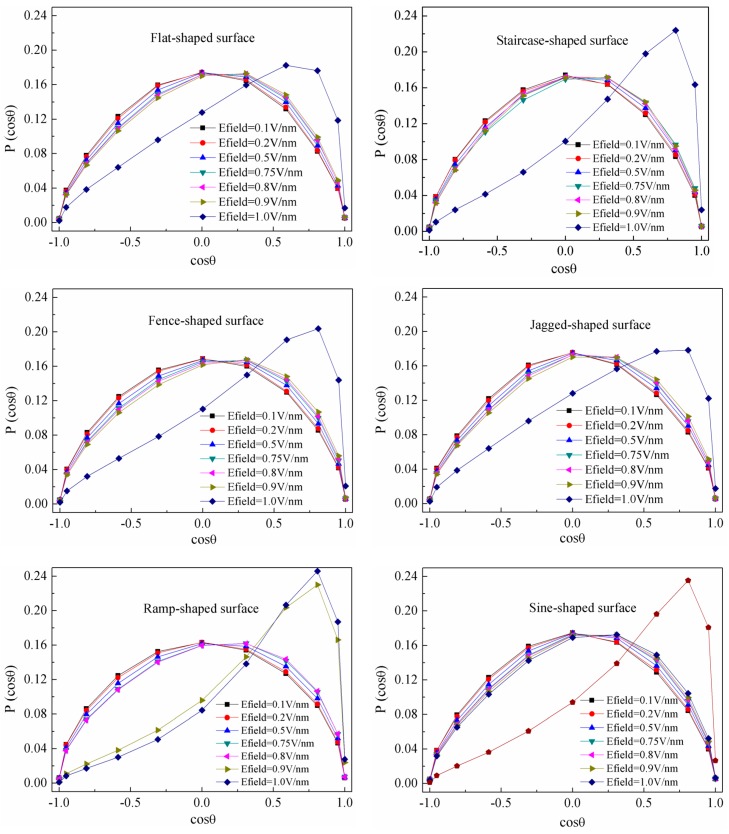
Distribution of average polarizations of water molecules under different electric field.

Water molecules have a tendency to self-arrange under the influence of an external electric field. Near the solid-liquid interface, the movement of water molecules is determined by the competition between the external electric field force, solid-liquid intermolecular forces and liquid-liquid intermolecular forces. In particular, the roughness of the solid surface makes the competition mechanism more complex. From the simulation data, the average polarization of water molecules along the direction perpendicular to the surface is calculated and the results are shown in Figure 11, where *θ* is the angle between the dipole moment vector of water molecules and the *y*-axis (*y*^+^). It was found that water molecules are in a disordered structure with water orientation distributions symmetric and broad at weak electric field *E* < *E*_c_. Here, *E*_c_ is the characteristic electric field and it is about 0.8 V/nm, being consistent with the literature [15]. When the electric field strength increases up to 0.9 V/nm, half the water dipoles in the case of the ramp-shaped surface exhibit an angle smaller than 30 degrees, while in the other cases, water dipoles were still distributed randomly, indicating that the restriction from ramp-shaped surface is smaller than others. When the applied electric field strength increases to *E_y_* = 1.0 V/nm, external electric field force is greater than the other intermolecular forces. Electric field force plays a more decisive role in the rearrangement of water molecules, with 50% or more water dipoles exhibiting a preferential orientation parallel to the direction of the electric field, which contributes to the formation of an ordered water structure. Accordingly, the electro-wetting behavior of a water droplet on a solid substrate is controlled by a coupling effect among water, solid intermolecular forces and the electric field.

## 4. Conclusions

Molecular dynamic simulation was carried out to investigate the wetting behaviors of a water nano-droplet on a rough silicon solid surface under perpendicular electric fields. Six types of roughness were constructed to explore the complex wetting phenomenon of the nano-droplet. The contact angle, obtained from fitting the density counter using an ellipsoidal equation, characterized the wetting properties. In the absence of an electric field, the static contact angles of a nano-droplet on different rough solid surfaces were analyzed and it was found that contact angle showed a slight decrease and a small difference with the increase of roughness factor. Meanwhile for the ramp-shaped surface, it tends to increase a little because the appearing bottom space reduces the interaction between water droplet and solid substrate. After an electric field was applied, water molecules realign themselves under the coupling effect of electric field force and intermolecular forces, accompanied by the increase of contact angles as the electric field strength increases. With the further increase of electric field strength, the nano-droplet was stretched to become column-shaped at a threshold value of the electric field. The threshold value is 0.9 V/nm for a ramp-shaped surface, while it is 1.1 V/nm for a sine-shaped surface. Thus, the wetting behaviors of a droplet on a rough solid surface is a result of the competition among the electric field force, the interaction force between the solid surface and the water molecule, and the interaction force between the water molecules.

## Figures and Tables

**Table 1:** Parameter of atoms for potential function.

Atom	*σ* (nm)	*ε* (kJ/mole)	*q* (e)
**O**	0.316	0.6502	−0.8476
**H**	0	0	0.4238
**Si**	0.3615	1.26	0.0

**Table 2:** Roughness Factors and Numbers of silicon atoms in solid substrates.

Rough Shape	Flat	Staircase	Fence	Jagged	Ramp	Sine
**Roughness factor (r)**	1.00	1.40	1.60	1.17	2.11	1.13
**Number of atoms**	13,206	12,030	12,030	11,993	9531	11,442

## Supplementary Materials

The following are available online at http://www.mdpi.com/2079-4991/8/5/340/s1, Figure S1: Equilibrium snapshot of water nano-droplet on silicon substrate (jagged-shaped surface) under electric field (*E_y_* = 0.1, 0.2, 0.5, 0.75, 0.8, 0.9 V/nm); Figure S2: Equilibrium Snapshot of water nano-droplet on silicon substrate (sine-shaped surface) under electric field *E_y_* = 1.1 V/nm.
