# Supplementary material for: Wetting Behaviors of a Nano-Droplet on a Rough Solid Substrate under Perpendicular Electric Field
Source: Nanomaterials (Basel). 2018 May 17;8(5):340. doi: 10.3390/nano8050340 (PMC5977354; doi:10.3390/nano8050340)
Supplement: Supplementary file 1 [file nanomaterials-08-00340-s001.pdf]

Electronic Supplementary Material for " ***Nanomaterials*** "

Supplementary information for:

Wetting Behaviors of a Nano-droplet on a Rough Solid Substrate under Perpendicular Electric Field

Fenhong Song [1], Long Ma [1], Jing Fan [1,*], Qicheng Chen [1], Lihui Zhang [2] and Ben Q. Li [3,*]

[1] School of Energy and Power Engineering, Northeast Electric Power University, Jilin 132012, China; fenhongsong@neepu.edu.cn (F.S.); ml158484015@126.com (L.M.); chenqicheng2010@hotmail.com (Q.C.)

[2] Key Laboratory of Special Purpose Equipment and Advanced Processing Technology, Ministry of Education, Zhejiang University of Technology, Hangzhou 310014, China; lhzhang@zjut.edu.cn

[3] Department of Mechanical Engineering, University of Michigan, Dearborn, MI 48128, USA

* Correspondence: crystalfan@neepu.edu.cn (J.F.); benqli@umich.edu (B.Q.L.); Tel.: +86-151-4424-8188 (J.F.); +01-313-593-5241 (B.Q.L.)

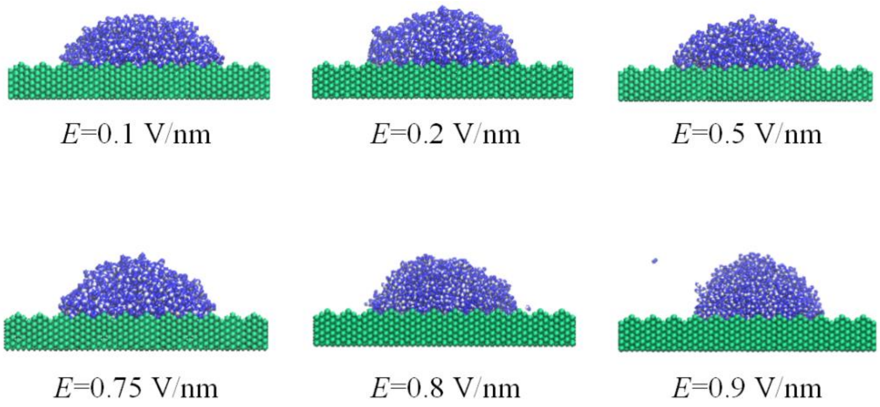

Figure S1. Equilibrium snapshot of water nano-droplet on silicon substrate (ramp-shaped surface) under electric field (E_y = 0.1, 0.2, 0.5, 0.75, 0.8, 0.9 V/nm)

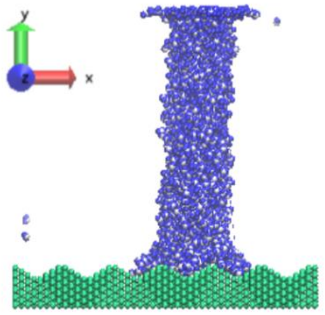

Figure S2. Equilibrium Snapshot of water nano-droplet on silicon substrate (sine-shaped surface) under electric field E_y = 1.1 V/nm
